# First-Principles Study of Adsorption of Pb Atoms on 3C-SiC

**DOI:** 10.3390/ma16206700

**Published:** 2023-10-16

**Authors:** Michal Komorowicz, Kazimierz Skrobas, Konrad Czerski

**Affiliations:** 1 National Centre for Nuclear Research, ul. A. Soltana 7, 05-400 Otwock-Swierk, Poland; michal.komorowicz@ncbj.gov.pl (M.K.); kazimierz.skrobas@ncbj.gov.pl (K.S.); 2 Institute of High-Pressure Physics of the Polish Academy of Sciences, ul. Sokolowska 29/37, 01-142 Warsaw, Poland; 3 Institute of Physics, University of Szczecin, ul. Wielkopolska 15, 70-451 Szczecin, Poland; 4 Institut für Festkörper-Kernphysik GmbH, Leistikowstraße 2, 14050 Berlin, Germany

**Keywords:** first-principles study, density functional theory, adsorption of Pb atoms, 3C-SiC

## Abstract

Changes in the atomic and electronic structure of silicon carbide 3C-SiC (β-SiC), resulting from lead adsorption, were studied within the density functional theory. The aim of the study was to analyze the main mechanisms occurring during the corrosion of this material. Therefore, the investigations focused on process-relevant parameters such as bond lengths, bond energies, Bader charges, and charge density differences. To compare the magnitude of the interactions, the calculations were conducted for three representative surfaces: (100, 110, and 111) with varying degrees of lead coverage. The results indicate that chemisorption occurs, with the strongest binding on the hexagonal surface (111) in interaction with three dangling bonds. The adsorption energy rises with increasing coverage, especially as the surface approaches saturation. As a result of these interactions, atomic bonds on the surface weaken, which affects the dissolution corrosion.

## 1. Introduction

Silicon carbide semiconductors, known for their robustness and chemical resistance, are broadly applied across diverse industries such as electronics, automotive, aerospace, and power generation. The research is focused on the cubic form of silicon carbide, 3C-SiC (β-SiC), which exhibits a zinc-blende structure with uniform bond lengths. This arrangement is the product of two interconnected face-centered cubic lattices, offset along the diagonal by a quarter of its length [1]. Both the material’s crystal composition and its high binding energy lead to exceptional stability and unique properties. These include hardness, superior thermal conductivity, resistance to radiation, and high temperatures, all of which make it suitable for deployment in extreme conditions like those found in fusion [2] and fission reactors [3]. For instance, it serves as a shielding material in TRISO fuel [4]. Furthermore, SiC should also be the main construction material of the new generation of high-temperature nuclear reactors such as the Dual Fluid Reactor [5]. In this reactor, liquid uranium–chromium eutectic will be used as fuel which will flow through a large number of closely placed SiC tubes. Liquid lead is expected to function as a coolant that flows along the SiC tubes and removes the produced heat from the reactor core. Thus, the corrosive interaction between liquid metals and SiC ceramics under the irradiation of high-energy neutrons will likely be one of the most important limiting factors that determine success of future reactor constructions—see also a similar design of a heat pipe microreactor [6].

Utilizing first-principles methods to observe shifts in electronic factors that dictate bond formation and breakage allows for the determination of adsorption energies, and investigation of reaction mechanisms and resulting structural changes, which define the macroscopic effects. It has been shown that the density functional theory can provide corrosion indicators for chosen ceramic materials such as the adsorption energy of corrosive atoms and corresponding charge density differences predicting the experimental results [7]. No similar studies have been identified for 3C-SiC, so far. Determining these effects, combined with studying the effects related to vacancies, will allow the intensity of this material’s dissolution corrosion to be determined in future studies. On the other hand, the exploration of interactions between lead and silicon carbide and the resulting changes in the electron structure of the surface may hold significant implications for various domains. These include, besides corrosion science, material science and nanotechnology [8], as well as less obvious areas such as lead detection and removal [9]. This study investigated the adsorption of lead atoms on three different reconstructed [10,11] 3C-SiC surfaces (100, 110, 111), with either carbon or silicon terminations. Tests were conducted for various monolayer (ML) coverages. By comparing the scale of effects for various surfaces, the most resistant one can be identified.

## 2. Method

The present study employs a computational methodology based on Density Functional Theory (DFT), implemented in the Plane-Wave Self-Consistent Field (PWscf) programs, a component of Quantum ESPRESSO—open-source software for quantum computations [12,13]. The pseudopotentials employed [14] are based on the Projector Augmented-Wave (PAW) method. The electron exchange–correlation effects were treated within the generalized gradient approximation (GGA) utilizing the Perdew–Burke–Ernzerhof (PBE) [15] exchange–correlation functional. The choice of method was motivated by its use by other researchers in similar research topics [7,16,17]. For all calculations, the wavefunctions cutoff was set to 50 Ry, and the charge density cutoff was set to 350 Ry. The lead potential was generated using fully relativistic calculations, while other potentials were generated with scalar-relativistic calculations. Noncollinear spin polarization was included in the calculations to account for spin-orbit coupling. Gaussian smearing of state occupations was utilized with a factor of 0.01 Ry. The electron convergence threshold was set to 10^−6^ Ry.

The lattice parameter was determined using the Birch–Murnaghan isothermal equation of state [18] for a periodically replicated single cell. The calculated constant (4.379 Å) aligns with the simulation [19] and the experimental results [20] (4.348 Å), which were subsequently used to create the adsorption models [21,22]. All simulations utilized periodic boundaries to create a slab model, integrating a vacuum region of three lattice constants in the z-direction to reduce interactions with the image of the next slab’s underside. Truncation of the crystal’s bulk structure results in the appearance of unbalanced forces acting upon the atoms. Surface reconstructions were executed to locate their new equilibrium positions. To ensure a proper transition to the bulk crystal, the two lowermost layers were fixed to the bulk coordinates. The dangling bonds of the bottom surface were saturated with hydrogen atoms, which were fixed in the obtained positions for the adsorption calculations. Relaxations were conducted until the forces acting on the atoms were below 0.02 eV/Å and total energy changes were below 0.13 meV.

Various coverages were examined by placing lead adatoms on supercells composed of 2 × 2 and 3 × 3 cells. These configurations correspond to a monolayer (ML) coverage of 1/4 and 1/9 ML. Due to the importance of mutual interaction between lead atoms at higher atomic packing, investigations were conducted on configurations with two or more lead atoms on a 2 × 2 supercell, which corresponds to 1/2 ML, 3/4 ML, and 1 ML. For the integration of the Brillouin zone, k-point samplings of 6 × 6 × 1, 3 × 3 × 1 Monkhorst–Pack grids were applied accordingly; for other cases, they were proportionally adjusted. High-symmetry initial adsorption sites (as applicable) were considered: T (on top), B (bridge), SB (short bridge), LB (long bridge), and H (hollow which for the (110) surface stands for on-top atom and for (111), fcc for the on-top atom in the lower layer and hcp above the hollow site). In post-processing, the charge density difference (CDD) was determined and the charge transfer from lead to the SiC surface was analyzed using Bader charge analysis [23]. CDD describes the disparity between the charge density of a system and a reference configuration and adatom [24]. With this method, alterations in charge distribution can be visually represented.

The adsorption energy *E_Ad_* is defined by the following equation:

*E*_*Ad*_ = (*E*_*SiC*+*Pb*_ − *E*_*SiC*_ − *N*
*E*_*Pb*_)/*N*

where *E_SiC+Pb_* is the total energy of a slab with an adsorbed lead atom, *E_SiC_* is the energy of the clean slab, and *E_Pb_* is the energy of an isolated lead atom. *N* denotes the number of adatoms. 

## 3. Results

### 3.1. (100) Surface

**Figure 1:**
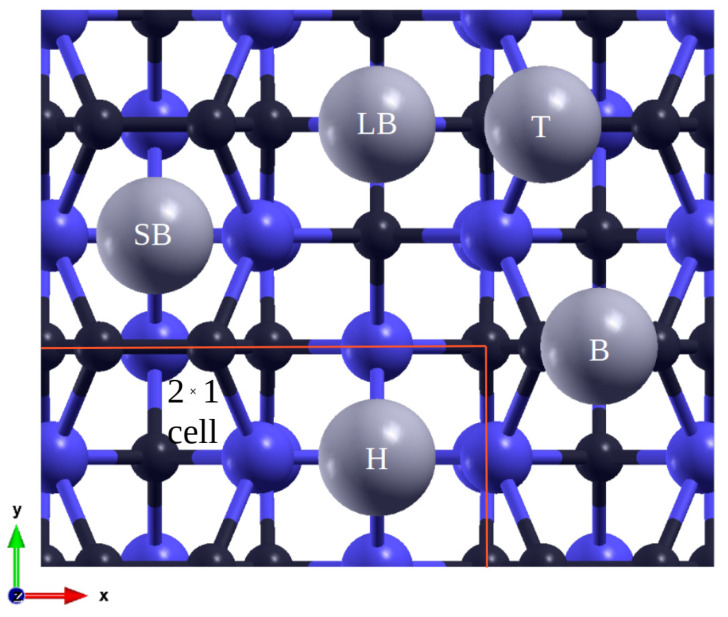
Initial adsorption positions of Pb (gray) atoms on SiC (100) surface, and Si (blue) C (black) atoms. T, on top; B, bridge; SB, short bridge; LB, long bridge; H, hollow between four dimers.

The (100) supercells consist of eight layers of atoms—four carbon, three silicon for the C-rich surface, and vice versa for the Si-rich surface. Both scenarios include a stabilizing hydrogen layer at the base. In order to facilitate the simulation of the formation of atomic pairs (dimers) during surface reconstruction, the samples were rotated by 45° along the *z*-axis, resulting in the arrangement shown in Figure 1. This adjustment does not impact the surface but simplifies calculations. While there exist multiple possible configurations [1], the linearly arranged dimers were reproduced in the case of the C-terminated (100) surface [25]. The formation of dimers reduces the number of dangling bonds and disrupts symmetries; hence, the smallest periodic space was composed of two cells in a 2 × 1 configuration. Consequently, coverages of 1/2, 1/4, and 1/16 ML were calculated for this case. The boundaries and adsorption sites are marked in Figure 1.

The derived carbon–carbon bond length was 1.374 Å, aligning closely with another study’s findings [26] (1.36). The on-top position was found to be unstable for all ML and transitioned to the bridge position. The short bridge position emerged as the most energetically favored with an average bond length of 2.47 Å and a donation of 0.89 electrons, which is the maximum among all the tested values. The results obtained are presented in Table 1. In the test of 1/2 ML with two atoms, the adatoms settled in positions of the bridge and non-adjacent long bridge but were not exactly aligned. A slight tilting led to a reduction in the distance between them, which measured 3.25 Å. Thus, the atoms form rows rather than a uniform lattice. The resulting energy was −2.16 eV per atom, which is remarkably close to the LB value for 1/4 ML; the bond lengths are slightly shorter than in that case and were 2.35 Å and 2.43 Å, respectively.

For the Si-rich (100) surface, the initial position was the ideal surface geometry. Considering the small energy difference from the configuration with dimers [27], this configuration was retained for further calculations after relaxation, during which the atoms moved solely vertically. For improved comparison, the 1/16 ML case was also computed. The atop site was unstable in 1/16 ML and, similarly, the SB position had the lowest energy and formed bonds that were 2.92 Å long, with a 0.33 charge transfer. Due to the symmetry of the relaxed lattice, here, SB is equivalent to H and LB to B. The obtained results are shown in Table 2.

In the tests involving two atoms, the adatoms, due to repulsion, shifted to the bridge positions, establishing an alternating rows arrangement and staying at the same height. The obtained energy per atom was −2.24 eV which is lower than the cases of 1/4 ML. Probably due to the occupation of the second, free bridge position in the cell. The bond length with silicon was 2.78 Å for both instances. The final case 3/4 ML resulted in the formation of two layers of lead for both cases and therefore, further tests were not conducted. The stable positions were as for the previous case with the second layer atom in the center of this square arrangement. The bond lengths are similar, but the deviations in the positions of the atoms on the surface are smaller. Thus, for these surfaces, the saturated monolayer coverage will be somewhere between 1/2 and 3/4 ML.

**Figure 2:**
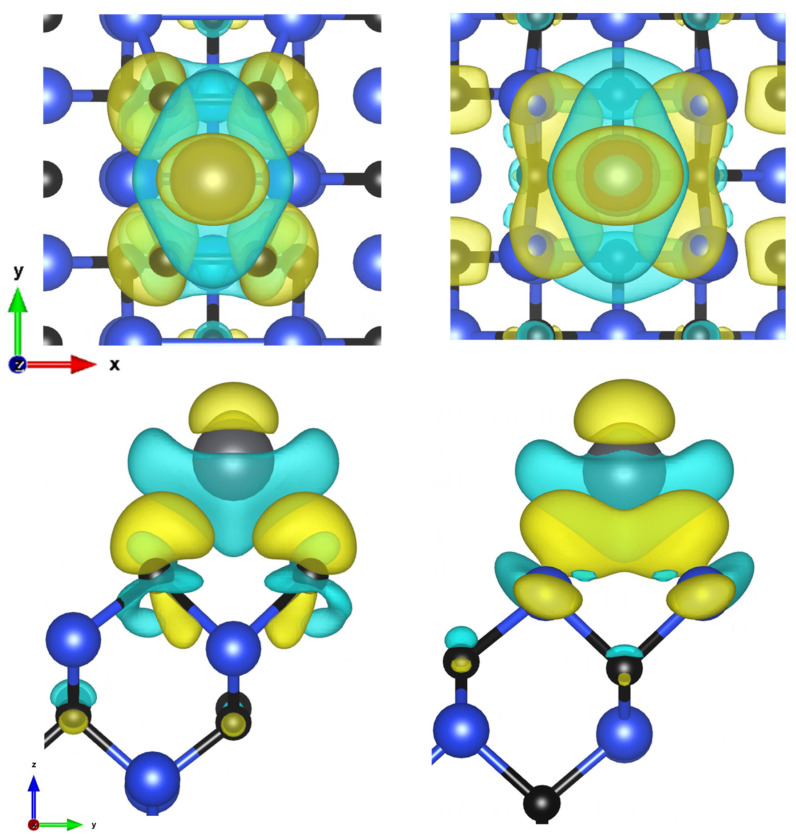
Charge density difference visualization of C-face and Si-face of (100) plane 1/4 ML and short bridge sites, in top and side views (isosurface values: 2.8 and 1.9 me/Å^3^).

In Figure 2 and the rest of the CDD of the graphs, the yellow color indicates the spaces in which the charge density increased, and the blue color in which it decreased. The charge is transferred to the neighboring carbon and silicon atoms, respectively. There was also a small transfer to the over atom and to the carbon atoms in the layers below.

### 3.2. (110) Surface

**Figure 3:**
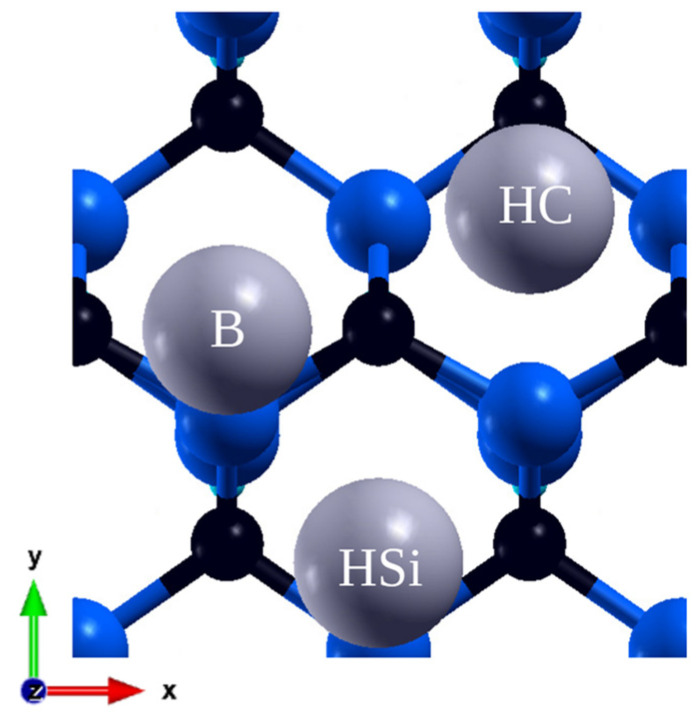
Final adsorption sites on (110) plane: hollow Si (HSi), hollow C (HC), bridge (B).

The surface made by the face diagonal plane (110) is nonpolar and low index. During the relaxation process, silicon atoms, as anticipated from materials with a similar structure, underwent both vertical and horizontal displacements, moving inward in the structure, while the C atoms positions remained almost unchanged [26]. A single cell contains two atoms on the surface, thus yielding smaller coverages of 1/4, 1/8, and 1/18 ML As in the previous case, higher coverages are created by adding more adatoms to the 2 × 2 supercell. This simulation space consisted of six layers of atoms and a hydrogen layer. In these calculations, there are two hollow sites, which almost overlap with an atom from the layer below, one being over Si and the second over C, as depicted in Figure 3. These two positions are the most stable, while others—the bridge, on top of Si and C—are unstable. The exception is the positions on the bridge in 1/8 ML. However, in the latter case, the atom did not retain its original position but moved to a new, unanticipated yet stable position (B) above the upper row of atoms, with an energy of −1.54 eV. This value is considerably smaller than the others so, given the instability of the other sites, it is plausible that after a significant increase in accuracy, it would have moved to the prevailing minima as well. The results are shown in Table 3.

**Figure 4:**
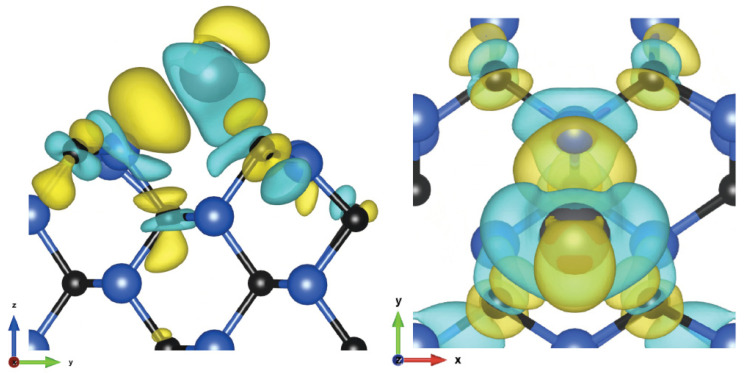
Charge density difference visualization of (110) plane 1/4 ML and hollow C site, in side and top views (isosurface value: 2.2 me/Å^3^).

For the hollow C site, lead donated 0.48 electrons, indicating a significant contribution from covalent bonding. Three bonds were formed, as shown in Figure 4, with lengths of 2.63 Å for the two carbon atoms and 2.76 Å for the silicon atom. In a simulation of 1/4 ML coverage, adatoms occupied HC positions, forming an alternating line. The adsorption energy is less than for other coverages, as seen also in case of the (100) surface. Bond lengths are shorter and was equal to 2.58 Å for C atoms and 2.75 Å for Si. In the next larger overlap of 3/8 ML, another vacant HC position was filled, directly next to the already occupied one; due to this short distance and adatom interaction, the adsorption energy increased. For the row of atoms thus formed, the distance from the C atoms increased to 2.83 Å and from Si, it increased slightly; the third lead atom remained almost unchanged. Only in this case, in a simulation with four adatoms, all of them remained on the surface. An additional atom also occupied the HC position and affected the atom directly next to it, similarly increasing its distance to the surface.

In this case, the charge was mainly transferred to the nearest silicon atom from which it was partially transferred to its neighboring carbon atoms, and also to a lesser extent, above the atom. Much less charge was transferred to the nearest carbon atoms because it was distributed between two of them.

### 3.3. (111) Surface

The final structure studied, with the surface formed by the body diagonal plane (111), consisted of nine atomic layers, including a layer of hydrogen and four layers each of C and Si. This surface occurs in two types with either one or three dangling bonds per atom on the surface; here, the first case is discussed. The supercell was reconstructed using a hexagonal cell. Although this arrangement resembles the α-SiC (0001) [7,28,29] surface, it has a different electronic structure which affects the length of the bonds to the next layer. Due to the hexagonal symmetries, only vertical displacements were observed in both the preparation and adsorption studies. The investigation covered three sites: on top, face-centered cubic (fcc) hollow, and hexagonal close-packed (hcp) hollow (over an atom from a deeper layer), all of which proved to be local minima.

**Figure 5:**
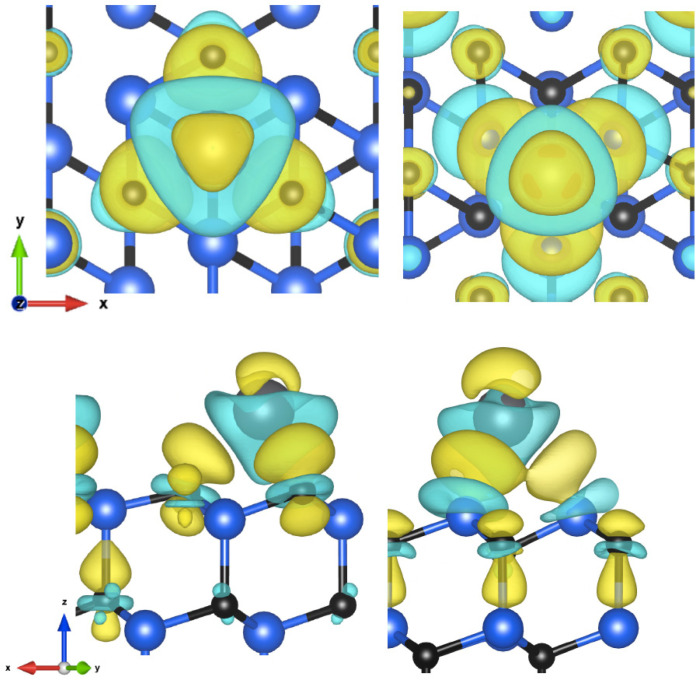
Charge density difference visualization of C-face (hcp) and Si-face (fcc) sites of (111) plane, in top and side views (isosurface values: 2.4 and 1.6 me/Å^3^).

For the C-face (111) surface, the hcp site, also the lowest of all obtained results, exhibited bonds of 2.52 Å with carbon. During the reaction, 0.82 electrons were exchanged. In both instances, the energies for the other hollow site were very close, differing by approximately 0.1 eV. In tests involving two atoms at 1/2 ML, the lead formed a honeycomb lattice, with atoms in on-top and hcp positions. The bond lengths were 2.29 Å and 2.57 Å, respectively, close to the values for 1/4 ML. The mean Pb-Pb distance was 3.52 Å. In the 3/4 ML, the atoms stabilized at the hcp, fcc, and on-top positions, forming a hexagonal lattice with a similar mean distance of 3.55 Å between lead atoms. The results are presented in Table 4. Among tests, for the (111) Si-face surface, the fcc site demonstrated the highest adsorption with a mean silicon bond length of 2.84 Å. However, there was a transfer of only 0.18 electrons, indicating a more covalent nature of the bond. For 1/2 ML, the same arrangement was formed, and the atoms occupied the same adsorption sites with bond lengths of 2.76 Å and 2.94 Å, respectively. The resulting distance between the lead atoms was 3.56 Å. The derived adsorption energies are very close to the corresponding average energy of these positions from 1/4 ML for both instances. In the 3/4 ML, during relaxation, the atoms passed through the same regular position, which in this case, turned out to be semistable. The final lattice consists of two deviated positions, on top and bridge, to form a less regular but single hexagonal layer, with distances ranging from 3.3 to 4.04 Å, with an average of 3.57 Å. The final 1 ML test resulted in a second layer of lead atoms in both cases. The three atoms on the surface occupied the same positions as for the 3/4 ML coverage, proving that the maximum monolayer coverage is between 3/4 and 1 ML. The results are presented in Table 5. The CDD diagram (Figure 5) obtained is almost identical to that presented in the detailed study of the SiC (0001) surface in [7] for the two outer layers of atoms. Also, the adsorption energies obtained are similar and show a comparable increasing trend; the Si-terminated surface also shows higher energies.

In the C-face, the transferred charge mainly accumulated around the nearest carbon atoms but also around the carbon atom of the lower layer farther from the adatoms, and to a lesser extent directly above the atom. In the second case, the charge was transferred to neighboring silicon atoms from where it spread to the carbon atoms below. A small fraction was also located above the atom.

**Figure 6:**
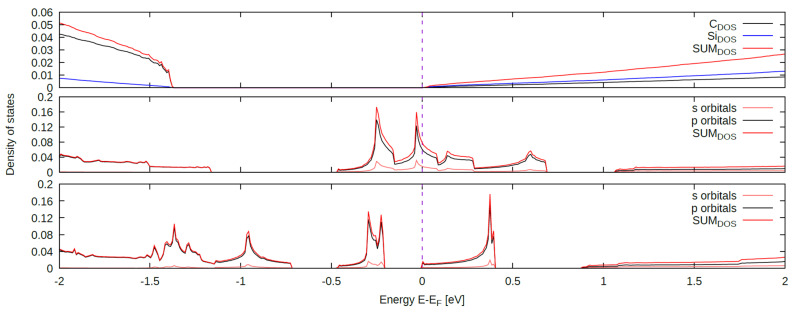
Density of states of the bulk crystal (**upper part**), the crystal (111) surface Si-face (**middle part**), and the surface with an adatom in the fcc site (**lower part**).

The graph (Figure 6) displays the density of states for the studied structures. The first graph of the bulk crystal structure shows a characteristic energy gap between the valence and conduction bands. Due to the nature of the utilized pseudopotentials, this energy gap appears smaller than its actual value. The subsequent chart illustrates the significance of unbound electrons—dangling bonds from the hybridization of the p orbitals of surface atoms—for surface stability. The energy states cross the Fermi level, causing the material to lose its semiconductor properties. The third graph indicates that the structure with an adatom is relatively stable. Si atoms tend to release electrons more easily than C atoms, causing additional electrons to accumulate over the Si atoms, which in turn, reduces the surface stability.

## 4. Discussion and Conclusions

The adsorption of lead was examined on three representative 3C-SiC surfaces with different coverage levels. Our findings revealed that only a subset of the probed positions exhibit stability. Additionally, a previously unidentified adsorption position was discovered. No studies have specifically addressed the adsorption of Pb on 3C-SiC. Instead, more attention has been directed towards the intercalation of this metal either on or beneath a graphene layer on SiC [30,31], driven by the potential to modify its properties through spin-orbit coupling. Research on 6H and 4H-SiC [7,16,32] also attracts attention due to their simpler production processes and greater availability. Unfortunately, the differences in configurations allows only for approximate comparisons of trends. Referring to [17], where a single SiC layer was explored, the bond length with Si (2.835 Å) closely aligns with our recorded value (2.84 Å) for a corresponding hcp position on the Si-face (111). Notably, the energy in this context is significantly lower, a variation likely attributable to the charge transfer extending to deeper layers. This bond length is also consistent with the (2.79 Å) measurement from [16] for the surface of 6H-SiC, and similarly for the fcc site.

The binding energies for all examined positions suggest chemisorption. It was observed that, as coverage increases, the binding energy typically rises as a result of adatom interactions, which can be observed especially for cases with smaller distances between adsorption sites. The exceptions are the cases of (100) for 1/2 ML coverage and (110) for higher coverages, which can be explained by the occupation of successive low-energy positions that are, however, distant enough to have little effect on the neighboring adatom. Importantly, no reactions leading to Si-C or C-C bond breakage were observed. This pattern may explain the generally weak interaction observed at the macroscale [33,34], where minimal or no response has been reported. As can be observed from the charge density difference graphs, in all cases, the lead atom transfers charge to the lattice, which predominantly accumulates around carbon atoms. This is due to the fact that carbon has the highest electronegativity of 2.52 among the atoms in the simulation, compared to 1.82 for silicon and 1.56 for lead [35]. Consequently, in configurations where a bond with carbon exists, a larger charge transfer from lead is observed, resulting in a relatively higher binding energy in most cases, with silicon in the crystal further enhancing this effect [36]. Charge accumulation between the lead atom and atoms on the surface leads to the weakening of the Si-C bond. This consequently promotes vacancy formation, which in turn leads to dissolution corrosion [7]. To assess potential differences in the progression of this process compared to α-SiC, further studies are needed. The explored cases of 1/2 ML and higher—an adsorption involving two or more lead atoms, as opposed to one [37]—underscore the significance of Pb-Pb interaction. Owing to this interaction, the most energetically favorable positions could be two distinct positions or their slight distortion, rather than stable positions in isolation, which cannot be studied with a single atom. The strongest bonds formed on the (111) surface at the hcp hollow site, where three bonds with carbon atoms yielded an energy of −3.64 eV. For the other instances, considering the lowest coverage, where a large distance Pb-Pb interaction is minimal, these include the fcc hollow position for (111) with an energy of −3.6 eV, hollow C for (110) with an energy of −2.36 eV, and the short bridge for both (100) surfaces, with energies of −2.33 and −2.53 eV, respectively. This pattern shows that the adsorption energy is a result of the specific arrangement of atoms exposed to interactions and their neighbors rather than just their surface energies [38]. Carbon attracts electrons more strongly, while silicon can transfer charges to three more carbon atoms, which is clearly visible in the case of (110). The lower binding energy in the (100) C-face is caused by the formation of dimers which results in a reduced number of dangling bonds. The results show that positions where there is interaction with one or two dangling bonds are much weaker or unstable, as opposed to stable positions held by three or four such bonds.

In conclusion, the first-principles study presented here may provide a basis for further qualitative analysis of the liquid lead-induced corrosion mechanism on SiC structural components of future advanced nuclear reactors in combination with thermodynamic methods, as recently reviewed by C.D. Taylor and H. Ke [39]. Considering the highest coverages examined, the adsorption energy is ranked in the order of (111) C-face < (111) Si-face < (110) < (100) C-face < (100) Si-face, indicating that the (100) surface will be least susceptible to lead exposure. Direct interaction of lead with carbon atoms, which possess a higher electronegativity, results in a higher charge transfer. This study showed that dangling bonds significantly affect the electronic structure and properties of the material and are determining factors for the adsorption process, but bonds to one or two of them are considerably less stable. Charge displacement weakens bonds in the lattice, promoting vacancy formation; the presented CDD graphs visually demonstrate sites of charge accumulation. Our results also show importance of the lead–lead interaction for the adsorption process and finding the most energetically favorable position for that.

## Figures and Tables

**Table 1:** Adsorption energies in eV for C-terminated (100) surface.

Adsorption Site	1/16 ML	1/4 ML	1/2 ML	3/4 ML
Short bridge (SB)	−2.33	−2.15		
Long bridge (LB)	−2.09	−2.16	−2.16	−2.04
Bridge (B)	−1.92	−1.83	−2.16	
Hollow (H)	−2.05	−2.23		

**Table 2:** Adsorption energies in eV for Si-terminated (100) surface.

Adsorption Site	1/16 ML	1/4 ML	1/2 ML	3/4 ML
Short bridge (SB)	−2.53	−2.19		
Top (T)		−1.09		
Bridge (B)	−2.23	−1.88	−2.24	−1.83

**Table 3:** Adsorption energies in eV for (110) surface.

Adsorption Site	1/18 ML	1/8 ML	1/4 ML	3/8 ML	1/2 ML
Hollow Si (HSi)	−2.05	−1.99			
Hollow C (HC)	−2.36	−2.34	−2.81	−2.46	−2.34

**Table 4:** Adsorption energies in eV for C-terminated (111) surface.

Adsorption Site	1/9 ML	1/4 ML	1/2 ML	3/4 ML	1 ML
Top (T)	−2.65	−2.56	−3.02	−2.72	−2.39
fcc Hollow	−3.51	−3.39		−2.72	−2.39
hcp Hollow	−3.64	−3.59	−3.02	−2.72	−2.39

**Table 5:** Adsorption energies in eV for Si-terminated (111) surface.

Adsorption Site	1/9 ML	1/4 ML	1/2 ML	3/4 ML	1 ML
Top (T)	−2.71	−2.43	−2.93	−2.77	−2.36
fcc Hollow	−3.6	−3.53			
hcp Hollow	−3.49	−3.45	−2.93	−2.77	−2.36
